# In Vitro Degradation of Pure Magnesium―The Effects of Glucose and/or Amino Acid

**DOI:** 10.3390/ma10070725

**Published:** 2017-06-29

**Authors:** Yu Wang, Lan-Yue Cui, Rong-Chang Zeng, Shuo-Qi Li, Yu-Hong Zou, En-Hou Han

**Affiliations:** 1 College of Materials Science and Engineering, Shandong University of Science and Technology, Qingdao 266590, China; tadyzwangyu@163.com (Y.W.); cuilanyue2010@126.com (L.-Y.C.); 2 College of Chemical and Environmental Engineering, Shandong University of Science and Technology, Qingdao 266590, China; zouyh69@126.com; 3 National Engineering Centre for Corrosion Control, Institute of Metals Research, Chinese Academy of Sciences, Shenyang 110016, China; ehhan@imr.ac.cn

**Keywords:** magnesium, XPS, polarization, acid corrosion, interface

## Abstract

The influences of glucose and amino acid (L-cysteine) on the degradation of pure magnesium have been investigated using SEM, XRD, Fourier transformed infrared (FTIR), X-ray photoelectron spectroscopy (XPS), polarization and electrochemical impedance spectroscopy and immersion tests. The results demonstrate that both amino acid and glucose inhibit the corrosion of pure magnesium in saline solution, whereas the presence of both amino acid and glucose accelerates the corrosion rate of pure magnesium. This may be due to the formation of -C=N- bonding (a functional group of Schiff bases) between amino acid and glucose, which restricts the formation of the protective Mg(OH)_2_ precipitates.

## 1. Introduction

Ageing of the global population has aroused public concern regarding health and biomedical materials and has generated huge demands for degradable implants and absorbable stents [1]. Magnesium (Mg) and its alloys have received much attention due to their good biocompatibility and biodegradability [2]. Mg is essential to the human body; it can promote bone healing and growth [3]. It is also an important component of 300 enzymes that act as catalysts [4]. The density and modulus of elasticity for Mg alloys are similar to that of the bones of body, which designate good mechanical compatibility and potential effectiveness to relieve stress blocking effects [5,6].

However, it is challenging to find successful biomedical applications of Mg alloys because of their rapid corrosion rate in the micro-environment of human body tissues [7]. These tissues contain inorganic ions (i.e., Cl^−^, HPO_4_^2−^, HCO_3_^−^, and Ca^2+^) as well as organic molecules (e.g., glucose, protein or amino acid) [8,9,10] that have a critical impact on the degradation behavior of Mg alloys.

To date, we lack a thorough understanding of the degradation mechanism of Mg alloys, although a considerable number of investigations involve the influence of alloying elements (i.e., Ca, Zn, Li) [4,6,11,12], secondary phases or intermetallic compounds [13,14,15,16], or other species and Tris-HCl buffer in biological solutions [17,18,19]. In most cases, in vitro corrosion tests have been performed in simulated body fluids (SBF) such as 0.9 wt. % NaCl aqueous solution, phosphate buffered solution (PBS) and Hank’s solution, in which only inorganic species (e.g., Cl^−^, HCO_3_^−^, HPO_4_^2−^ and H_2_PO_4_^−^) exist. The interaction between the alloy/solution interface leads to ionization of Mg. These metallic ions reacting with the anions (i.e., HCO_3_^−^, HPO_4_^2−^ and H_2_PO_4_^−^) of the solution result in the formation of precipitates, which have an important impact on the corrosion behavior of the alloys [20,21]. Our previous investigations [22] demonstrate that Cl^−^ ion increases the degradation rate; presence of bicarbonate and sulfate ions in saline solution accelerates the corrosion, while phosphate ions slow down the corrosion rate.

It is noted that there are numerous organic molecules such as glucose, amino acids and proteins in body fluids. The influence of the organic molecules on the degradation of Mg alloys has, however, been scarcely reported. Our previous study designates that the corrosion rate of pure Mg increases with the glucose content in physiological saline (0.9 wt. % NaCl) solutions because glucose rapidly transforms into gluconic acid, which decreases the solution pH and attacks the oxidation film of the pure Mg surface. In contrast, higher concentrations of glucose in Hank’s solutions result in a decrease in the corrosion rate. Moreover, glucose promotes the absorption of Cl^−^ ion on the pure Mg surface; but in Hank’s solution, glucose coordinates Ca^2+^ ion, facilitating the formation of Ca-P compounds and preventing corrosion on the Mg surface [23]. 

As biological macromolecules, proteins also play an important role in the corrosion process of Mg alloys. Protein molecules combine with metals on the surface of alloys by electrostatic attraction, hydrogen bonds and ionic bonds, which are all related to the electrostatic interactions [24,25]. The process is affected by many factors, such as solution composition, protein concentration, and the physical properties of the material itself. Adsorption and chelation are the main mechanisms by which proteins influence the degradation of Mg. When investigating the influence of proteins on the corrosion behavior of Mg alloys, there are many issues to consider. On the one hand, the presence of proteins reduces the corrosion rate of Mg and its alloys [26,27] due to the adsorption of protein and the formation of a protein layer on the surface of Mg alloys. Moreover, the higher the concentration of albumin, the higher the inhibitive effect. On the other hand, studies [28] also reveal that proteins accelerate the corrosion rate of Mg alloys. Yang et al. [29] has indicated that a higher concentration of foetal bovine serum (FBS) improves the corrosion rate. This result is ascribed to the loss of H^+^ from the -COOH group of the protein and the resulting change in the solution pH value. Proteins are composed of amino acids, and there are dozens of free amino acids in body fluid. It is, therefore, essential to better understand the influence of amino acids on the degradation of biomedical Mg alloys [30]. It has been reported that amino acids can promote the dissolution of the pure Mg surface [20]. Nevertheless, further investigations on the effect of amino acids on corrosion of Mg alloys are necessary. In particular, the coupling effect of glucose and amino acid on the degradation of biodegradable Mg alloys has not yet been elucidated. 

The present work aims to investigate the synergetic effect of glucose and amino acid on the degradation behavior of pure Mg and to provide further insight into the degradation mechanisms of biomedical Mg from a novel perspective.

## 2. Results and Discussion

### 2.1. Immersion Test

**Figure 1:**
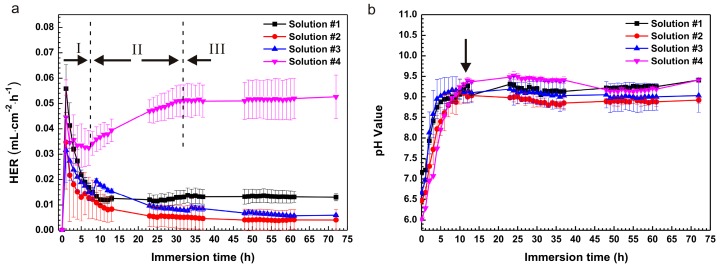
Curves of (**a**) hydrogen evolution rate (HER) and (**b**) pH values as functions of immersion time.

Figure 1a shows the hydrogen evolution rate (HER) of the pure Mg samples exposed to saline solutions with different amino acid and glucose contents at 37 ± 0.5 °C for 72 h. The samples showed a high HER at initial stage during the soaking experiments and a distinct drop with further immersion. It might be ascribed to the breakdown of the oxide film on the surfaces of the samples. At the first soaking of 2 h, HER in solution #1 was the highest; after 2 h of immersion, HER in solution #4 was the highest. Moreover, HER in solution #2 and #3 showed a sudden and small increase after immersions of 5 h and 9 h, respectively. The reason for this remained unclear. As a result, HER in solution #2 and #3 fell again to their normal levels. The lowest HER in solution #2 indicated that L-cysteine was beneficial for the inhibition of corrosion. The lower HER in solution #3 compared to solution #1 after 20 h was due to the diminishment of glucose because glucose transformed into gluconic acid in saline solution and promoted corrosion on the pure Mg surface [23]. Furthermore, HER in solution #4 slightly decreased at initial phase (8 h of immersion), then increased until 32 h of immersion and finally remained at a stable level. This situation was attributable to the coupling effect of glucose and L-cysteine, and might cause the formation of new chemicals and acceleration of the corrosion. Consequently, HER remained stable due to the depletion of glucose and L-cysteine and the formation of a protective film. 

Generally, HER is related to solution pH value. The changes in solution pH during the immersion are shown in Figure 1b. The initial pH of the solutions increased in the following order: solution #4 < solution #2 < solution #3 < solution #1. Obviously, solution #1 was neutral; solutions #2–#4 were acidic due to the presence of glucose and/or L-cysteine. In addition, glucose was changed into gluconic acid. The dissolution of Mg can dramatically enhance the pH value for all of the samples. The curves of the pH value as a function of immersion time experienced two stages: one is an initial and steep rising stage; the other is a horizontal stage. It is noteworthy that in the initial stage with an immersion of 12 h, the pH value in solution #4 continuously increased from the lowest to the highest; it then declined slightly until 35 h of immersion; finally the pH of solution #4 became lower than that of solution #1 after 45 h of immersion. This scenario might be due to the dissolution of the Mg(OH)_2_ precipitates into soluble MgCl_2_ in solution #4 and the continual formation of Mg(OH)_2_ in solution #1. It is clear that the corresponding pH values had a remarkable impact on HER.

### 2.2. Electrochemical Experiments

**Figure 2:**
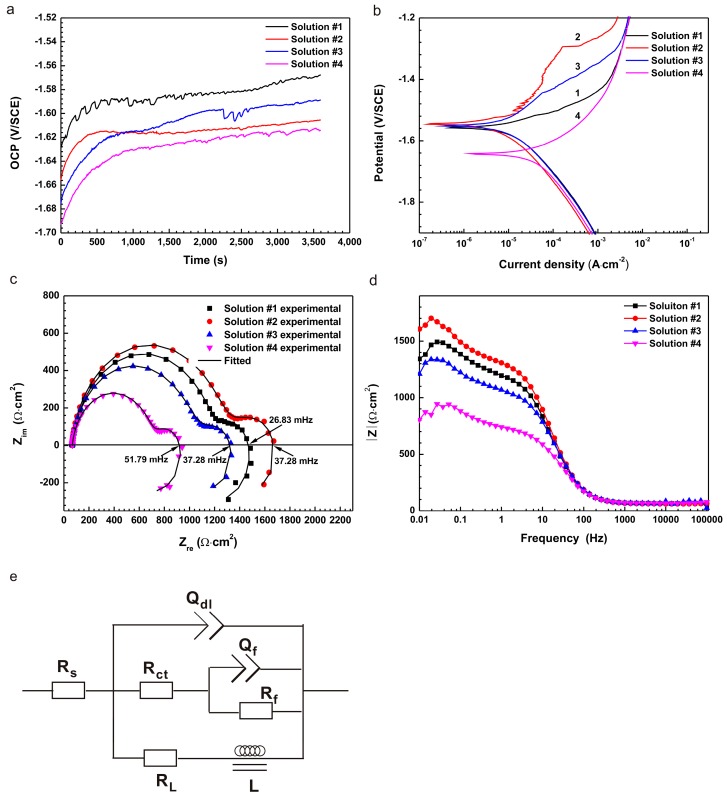
The plots of (**a**) open circuit potential (OCP); (**b**) polarization; (**c**) Nyquist (including the fitting curves) and (**d**) Bode curves; and (**e**) the equivalent circuits of the electrochemical impedance spectroscopy (EIS) spectra.

The corrosion behaviors of metals are also characterized by virtue of electrochemical tests such as open circuit potential (OCP), polarization and electrochemical impedance spectroscopy (EIS). As shown in Figure 2a, the increase in OCP indicated the formation of a corrosion product layer on the pure Mg surface. Note that the different contents of L-cysteine and glucose resulted in different corrosion potentials. Open circuit potential of the samples in solution #1 fluctuated frequently and exhibited a zigzag shape throughout the test, implying that it was difficult to reach a steady state for the formation and dissolution of the corrosion product film at the electrode/solution interface. The fall in the OCP curve in solution #1 can be attributed to the rupture of the corrosion product film. In solution #2, OCP started to stabilize at 500 s of immersion and only slightly decreased at approximately 700 s. Open circuit potential in solution #3 continuously rose, but there was a sudden drop at approximately 2200 s of immersion, which indicated the rupture of the surface film. In solution #4, there was also a slight fluctuation in the OCP curve at a later stage, which could be ascribed to the pitting corrosion that resulted from the coupling effect of L-cysteine and glucose [31]. 

The polarization curves (Figure 2b) indicated that the sample in solution #4 had the lowest *E*_corr_ and the largest corrosion current density (*i*_corr_). There was a clear breakdown potential (*E*_b_) in solution #2 and #3, and the *E*_b_ in solution #2 was higher than that in solution #3. The anodic curve in solution #2 designated a big passivity zone and self-repairing ability, denoting that a protective film formed on the Mg surface. The electrochemical parameters of polarization curves for pure Mg samples in the four solutions were listed in Table 1. The results indicated that the presence of L-cysteine gave rise to the lowest *i*_corr_ and the largest *R*_p_. That is, L-cysteine enhanced the corrosion resistance in solution #2 in comparison to that in solution #1. However, the presence of L-cysteine mixed with glucose in solution #4 resulted in the highest *i*_corr_. This meant that the synergetic effect of L-cysteine and glucose increased the corrosion rate of pure Mg in saline solution. 

Nyquist plots (Figure 2c) of the samples demonstrated one high-frequency capacitance loop, one low-frequency capacitance loop and an inductance loop. The low-frequency inductance loops appeared at the frequency of 37.28, 26.83, 37.28, and 51.79 mHz for solution #1, #2, #3, and #4 in turn. The diameter of the capacitance loop for pure Mg is the largest in solution #2, and the smallest in solution #4, demonstrating that the L-cysteine alone could improve the corrosion resistance; but the coupling effect of L-cysteine and glucose could decrease the corrosion resistance. This finding was in pronounced agreement with the results of HER and the polarization curves. 

To further clarify the corrosion characteristics of the samples, the EIS plots were analyzed based, on the equivalent circuits (R(Q(R(QR))(LR))) shown in Figure 2e [32,33,34]. The data were fitted using ZSimpWin 3.20 software (AMETEK, San Diego, CA, USA) and the fitting results were listed in Table 2. *L* and *R*_L_ represented inductance and resistance, respectively, which were used to describe the inductance loops at low frequency [31]. The existence of inductance loops indicated the occurrence of pitting corrosion. *R*_ct_ represented the charge transfer resistance and a higher *R*_ct_ value implied a lower dissolution rate of Mg substrate [32,35,36]. The *R*_ct_, confirming the results from the loop diameters, could be ranked in the order: solution #2 > solution #1 > solution #3 > solution #4. Moreover, the low-frequency impedance modulus ׀Z׀ is one of the parameters used to evaluate the corrosion resistance of different samples in Bode plots (Figure 2d). A larger ׀Z׀ indicates a better corrosion resistance. It could be observed that the results of ׀Z׀ were in keeping with Nyquist plots.

### 2.3. Surface Analysis

**Figure 3:**
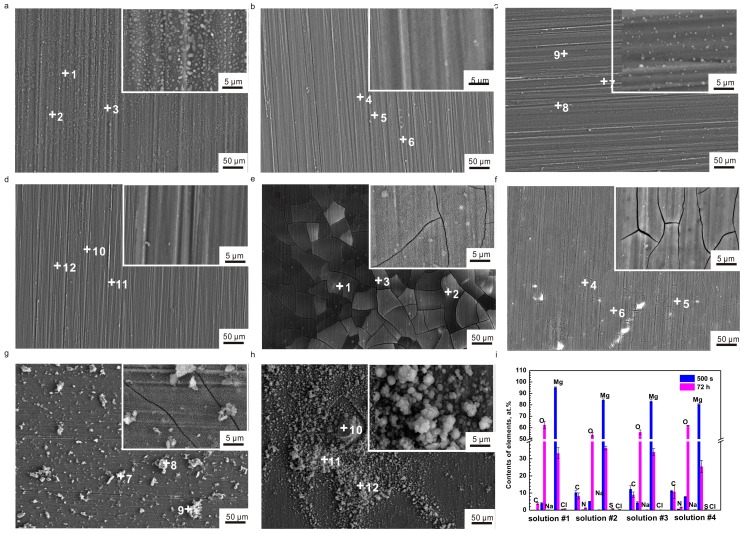
SEM morphology images of the pure Mg surface after immersions of 500 s: (**a**) solution #1; (**b**) solution #2; (**c**) solution #3; (**d**) solution #4; SEM morphology images of the pure Mg surface after immersions of 72 h: (**e**) solution #1; (**f**) solution #2; (**g**) solution #3; (**h**) solution #4; (**i**) contents of various elements probed by EDS for a comparison of 500 s with 72 h of immersion.

Figure 3a–d display the SEM morphologies of the samples after an immersion of 500 s. More granular corrosion products formed on the surface in solution #1 and fewer granular corrosion products formed on the surface in solution #3. No similar film or corrosion products occurred in solutions #2 and #4. Figure 3e–h show the SEM morphologies of the pure Mg samples immersed for 72 h. The SEM images revealed that the corrosion products formed on the surface of samples. There were numerous micro-cracks on the river-bed-like surface (Figure 3e), signifying the formation of a uniform corrosion product (Mg(OH)_2_) layer in solution #1. The number of micro-cracks obviously decreased, suggesting the formation of a dense corrosion product layer in solution #2 (Figure 3f). The corrosion morphology on the sample in solution #3 was similar to that in solution #1, besides the random dispersal of granular corrosion products (Figure 3g). Figure 3h shows that the most serious corrosion occurred on the samples exposed to solution #4. In addition, it could be found that there was an aggregation of granular corrosion products. 

From the results of EDS analysis (Figure 3i) for the samples after immersion of 500 s, the absence of C on the sample designated that no carbon dioxide in air dissolved in solution #1 at the initial immersion. However, the content of C was the highest in solution #3, second highest in solution #4, and the lowest in solution #2. This scenario implied that the organic compounds (amino acid and glucose) adsorbed on the surfaces and gave rise to the formation of a thin adsorption layer (Figure 3b–d). The amount of O element was very similar in solutions #1, #2 and #3 and was much lower than that in solution #4. The lower content of O and higher content of Mg indicated fewer Mg(OH)_2_ precipitates, or a weakened attack on the samples at the beginning of immersion. The concentrations of N in solutions #2 and #4 were 0.156 at. % and 0.56 at. %, respectively; and the S concentrations in solutions #2 and #4 were 0.16 at. % and 0.385 at. %, respectively.

In comparison to the EDS analysis of the samples after an immersion of 500 s, the presence of C might be due to the contaminants from air after 72 h of immersion in solution #1. The C element of the samples remarkably decreased in solution #3 and slightly decreased in solutions #2 and #4. The enhancement in both N and S contents in solutions #2 and #4 suggested the adsorption of more amino acids with time. Moreover, the higher concentration of N for the sample in solution #4 than solution #2 disclosed that glucose promoted the deposition of amino acids. The noticeable increase in O content suggested the formation of more corrosion product Mg(OH)_2_ over time. The decrease in Mg content, however, denoted the improvement in thickness of the corrosion product layer. There were very low content of Na and Cl elements, which were ascribed to the remaining from solutions.

**Figure 4:**
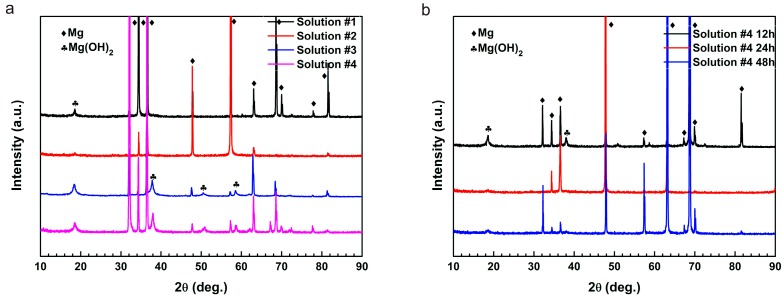
XRD patterns of pure Mg immersed in: (**a**) solution #1–4 for 72 h; and (**b**) solution #4 for 12–48 h.

Figure 4a shows the XRD patterns of the samples after immersions of 72 h. The peaks at 18°, 38°, 51°, and 59° corresponding to Mg(OH)_2_ confirmed that the corrosion product of Mg(OH)_2_ was on the surface of samples exposed in solutions except in solution #2. The XRD patterns demonstrated that L-cysteine obstructed the formation of the Mg(OH)_2_ film, and then inhibited the corrosion of pure Mg. However, the coupling effect of L-cysteine with glucose promoted the corrosion. To further clarify the corrosion behavior of the samples, we tested samples immersed in solution #4 for different lengths of time as shown in Figure 4b. Interestingly, the peaks of Mg(OH)_2_ after immersion for 12 h was the strongest, while it was reduced after immersions of 24 h and 48 h. It might be due to the dissolution of the corrosion product layer.

**Figure 5:**
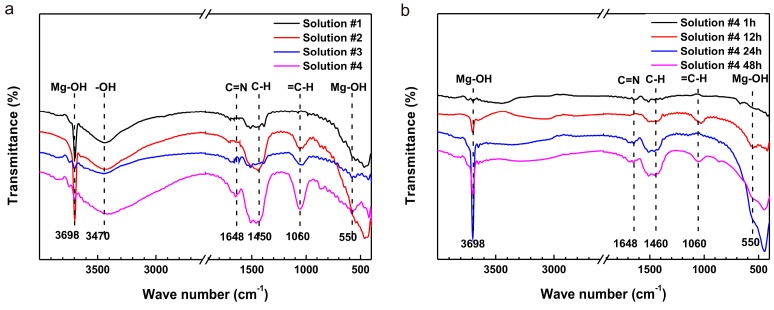
Fourier transformed infrared (FTIR) spectra of the samples in: (**a**) four solutions after an immersion of 72 h and (**b**) solution #4 after an immersion from 1 h to 48 h.

The Fourier transformed infrared (FTIR) spectra of the samples after an immersion of 72 h are shown in Figure 5. The absorption bands at 3698 cm^−1^ and 550 cm^−1^ corresponding to Mg-OH stretching vibrations revealed the existence of an Mg(OH)_2_ film. A band at approximately 1648 cm^−1^ could be ascribed to the bending vibration of an azomethine group (-C=N-) [37] resulting from the reaction between L-cysteine and glucose. The bands at 1450 cm^−1^ were typical vibration modes of C-H, which are included in L-cysteine and glucose molecules. Additionally, the bands at 1060 cm^−1^ could be designated to the in-plane bending vibration mode of =C-H, demonstrating that the vinyl in L-cysteine or glucose affected the surface corrosion of samples. The spectra of the samples with the immersion from 1 h to 48 h are shown in Figure 5b. The intensity of the -C=N- absorption band was gradual stronger indicating that the existence of a coupling effect would generate new compounds including the double bond.

**Figure 6:**
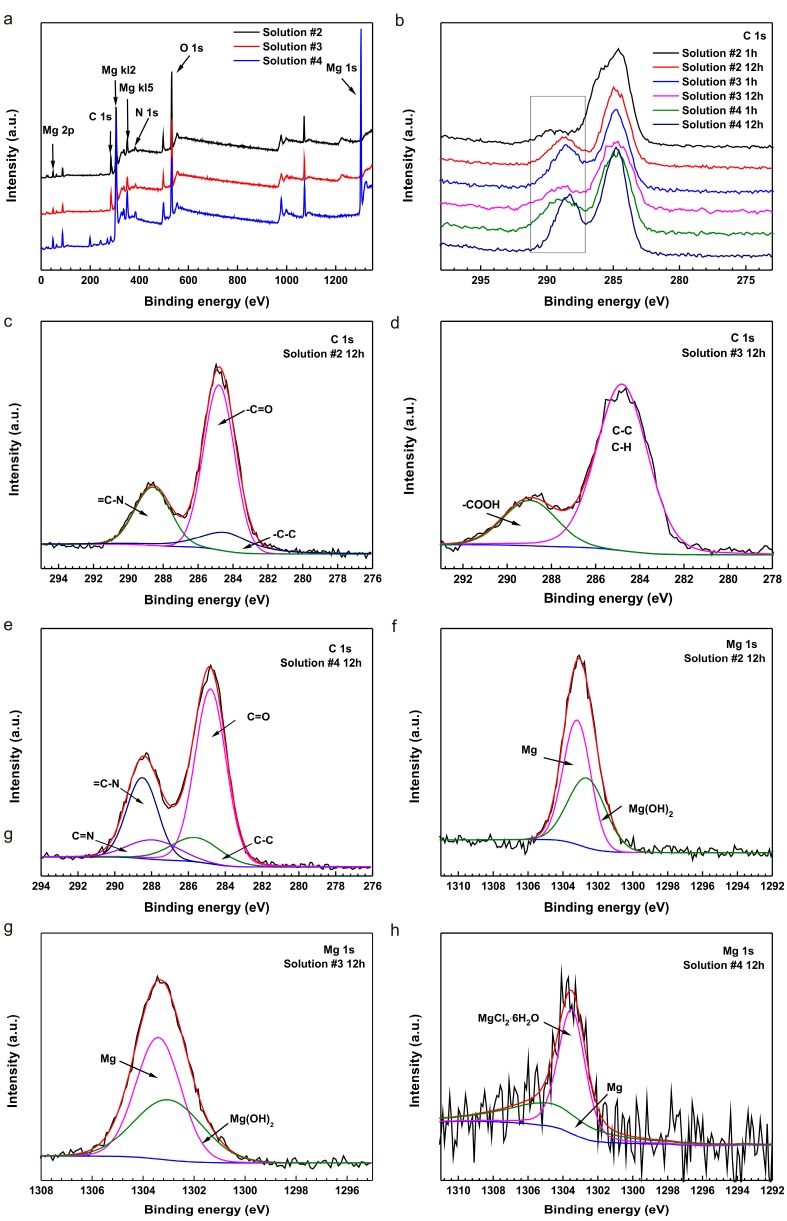
X-ray photoelectron spectroscopy (XPS) analysis of a pure Mg surface after immersion in solution #2 through #4 for various periods showing the (**a**) entire range of the binding energy survey and (**b**) C 1s spectra for sample surfaces after immersion in solution #2 to #4 for 1 h and 12 h; C 1s spectra of samples immersed in (**c**) solution #2, (**d**) solution #3 and (**e**) solution #4 for 12 h; Mg 1s spectra for samples after immersion (**f**–**h**) in solution #2 to #4 for 12 h.

To further demonstrate these results, X-ray photoelectron spectroscopy (XPS) analyses were performed on the samples immersed in 0.9% NaCl with L-cysteine and glucose for differential concentrations and times. Figure 6a shows the entire range of the binding energy survey, indicating the existence of Mg, C, O and N elements. For the element S, there was too little to be detected due to the short immersion time and only trace concentrations in the solutions. Figure 6b shows the C 1s spectra of the samples immersed in solutions #2 through #4 for 1 h and 12 h. It is interesting to note that a new signal appeared after immersion for 12 h (Figure 6b), implying that the L-cysteine and glucose were transformed into Schiff bases (a compound containing -C=N-). The results were in agreement with the results of the previous test. Interestingly, the selected peak at 288.90 eV of sample in solution #3 appeared after an immersion of 1 h but weakened after 12 h, implying that the glucose transformed to gluconic acid. In contrast, the selected peak intensity in solutions #2 and #4 got stronger with time. This result may be ascribed to the continuous generation of new substance.

Figure 6c–e designates the fitting curves of the C 1s spectra for the samples after immersions of 12 h in solutions #2 through #4. In solution #2 (Figure 6c), the C 1s spectra could be split into three peaks. The peak at 284.80 eV could be attributed to the -C=O- bond. The peak of 288.59 eV might be associated with the bond of =C-N-. When L-cysteine was mixed with glucose, the new peak at 287.90 eV could be associated with the -C=N- group (Figure 6e) [38]. Figure 6f–h [12] show the fitting curves of the Mg 1s spectra of the samples after immersion in solutions #2 through #4 for 12 h. With the addition of L-cysteine or glucose alone, the corrosion product mainly included Mg(OH)_2_ or MgO. But the coupling effect of L-cysteine and glucose led to a strong peak of MgCl_2_. Mg^2+^ might have combined with Cl^−^ to seed out on the surface.

## 3. Discussion

### 3.1. Influence of L-Cysteine on Degradation

It has been reported that the organic constituents of blood plasma include 30–55 g/L albumin [39]. The albumin molecules predominately comprise a variety of amino acids connected by peptide bonds. An amino acid usually contains an amino group (-NH_2_), a carboxyl group (-COOH), a central carbon (C) and a side chain as well [24]. In the neutral saline solution, albumin experiences a neutral-acidic transition and becomes negatively charged (isoelectric pH 4.7–4.9). Divalent Mg^2+^ ions can serve as bridging agents to promote the adsorption of albumin molecules on the surface of pure magnesium through electrostatic interaction. 

The previous investigations draw different conclusions and the data (*i*_corr_ and *R*_ct_) are summarized in Table 3. Liu et al. [40] indicated that protein (albumin) would form a film of protein to protect the samples and the capacity of protection was increased from 0 g/L to 10 g/L of albumin in 0.8 wt. % NaCl solutions. Wang et al. [28] came to the same conclusions in initial corrosion (SBF + 40 g/L albumin) of M1A alloys. It is proposed that the adsorption is much faster than chelation during the initial stage. Moreover, the chelation effect gradually becomes more significant than adsorption with time, leading to a higher corrosion rate. Nevertheless, Mueller et al. [41] showed that protein would accelerate the corrosion rate of pure Mg in PBS; the higher the concentration, the higher the corrosion rate. It was considered that the corrosion layer was probably not uniform and did not offer similar protective characteristics on the entire surface. Additionally, Yamamoto [20] indicated that amino acids would promote the corrosion process in E-MEM solution. This scenario was ascribed to amino acids, which reduced the barrier effect of the insoluble salt layer against dissolution of Mg and combined with Mg^2+^ to form a metal complex.

Mg is very active in aggressive saline solution. The Mg specimens quickly dissolved and released a massive amount of Mg^2+^ ion, alkaline hydroxyl anions and H_2_ gas [23]. This is consistent with the result of curves of HER and pH values. The chemical reactions are as follows:

Mg(s) → Mg^2+^(aq) + 2e^−^ (anodic reaction) (1)

2H_2_O + 2e^−^ → H_2_(g) + 2OH^−^(aq) (cathodic reaction) (2)

Mg^2+^(aq) + 2OH^−^(aq) → Mg(OH)_2_(s) (product formation) (3)

**Figure 7:**
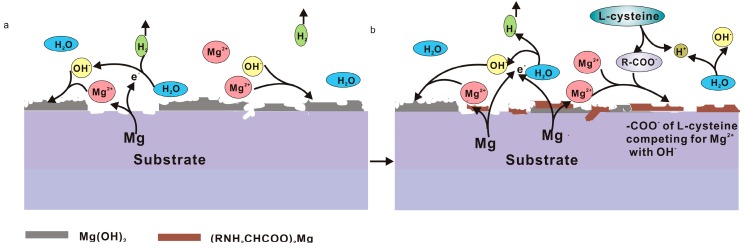
Schematic illustration of the corrosion process of pure Mg during immersion in solution #2: (**a**) initial corrosion mechanism and (**b**) corrosion during the later period.

Our data showed that L-cysteine could inhibit the corrosion of pure Mg in 0.9 wt. % NaCl solution. L-cysteine contains an amino group and a carboxyl group; it is electronegative in saline solution because the solution pH is higher than its isoelectric point (5.02). Hence, molecules of L-cysteine (HSCH_2_CH(NH_2_)COO^−^) probably combined with Mg^2+^ and were adsorbed on the surface of the samples to prevent further corrosion according to Reaction (4). Figure 7a–b illustrate the corrosion mechanisms.

Mg(OH)_2_ + 2HSCH_2_CH(NH_2_)COOH → (HSCH_2_CH(NH_2_)COO)_2_Mg + H_2_O (4)

Namely, a trace of amino acid prevented pure Mg from corrosion in saline solution due to the formation of a thin layer by adsorption. The influence of the content and the kinds of amino acid needs to be studied further.

### 3.2. Influence of Glucose on Degradation

**Figure 8:**
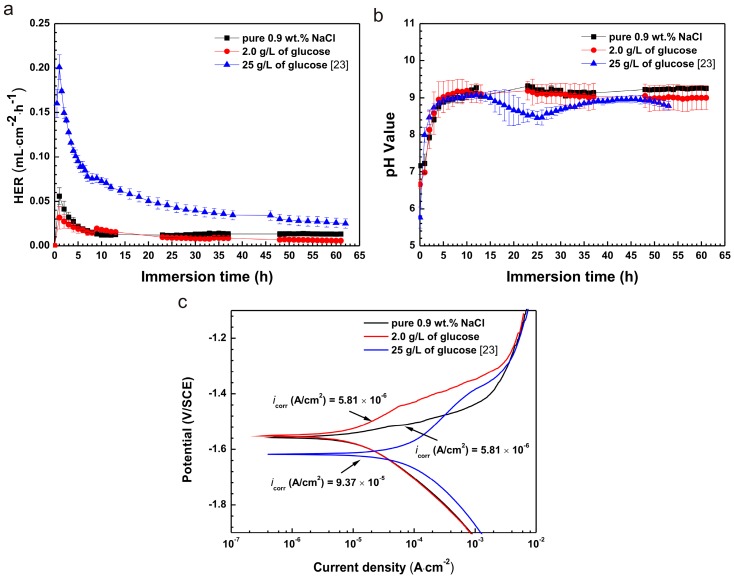
The comparisons of results between 2 g/L and 25 g/L glucose showing (**a**) HER; (**b**) pH value; and (**c**) polarization.

It is noteworthy that our previous work [23] showed that the presence of glucose (25 g/L) in saline solution accelerated the corrosion of pure Mg. In this study, however, it inhibited corrosion of pure Mg in the presence of glucose at a concentration of 2.0 g/L. The differences of HER are shown in Figure 8a. HER of the samples immersed in saline solution with a concentration of 2.0 g/L glucose were lower than those in 25 g/L glucose. Furthermore, the HER curves displayed a similar variation trend, increasing in initial time (after 1 h) and decreasing subsequently until reaching stability. The reason for the results may be concerned with the change in solution pH value.

As shown in Figure 8b, the pH values of solutions were all increased in initial immersions of 11 h. The values were, however, decreased at 20 h and 12 h in 2.0 g/L and 25 g/L, respectively. This likely resulted from different concentrations of glucose leading to the various pH values due to the shift of glucose into gluconic acid [23]. 

It can be seen in Figure 8c that the *i*_corr_ (5.81 × 10^−6^ A/cm^2^) of the sample immersed in saline solution with a concentration of 2.0 g/L was lower by one order of magnitude than that (9.37 × 10^−5^ A/cm^2^) in the 25 g/L solution. The differences observed across concentrations highlight what may be a critical effect regarding glucose content. Further studies are needed to more fully understand these potential effects.

### 3.3. Degradation Mechanism of the Coupling Effect of Amino Acids and Glucose

While it was mentioned above that the solutions with both L-cysteine and glucose increase the corrosion rate, amino acids react with glucose at room temperature in alkaline aqueous solutions [42,43]. Previous studies suggested that the -C=N- might result from the condensation of primary amine with carbonyl. The reaction follows as [30,44,45]:

R-NH_2_ + R’-COH → R-N=CH-R’ + H_2_O (5)

where R and R′ represent aliphatic and aromatic groups, respectively. Amino acids and glucose have R-NH_2_ and R′-COH groups, respectively. When combined, the chemical reaction between R-NH_2_ and R′-COH may be inevitable [30,46]. The protonation of the nitrogen atom of -CH=N- group could positively charge the molecules in aci`d solutions [24]. The nitrogen atom might be deprotonated in alkaline solutions (Figure 1b) and then react with positively charged Mg^2+^ ion. As a result, the corrosion rate of the sample might be accelerated as observed in solution #4.

**Figure 9:**
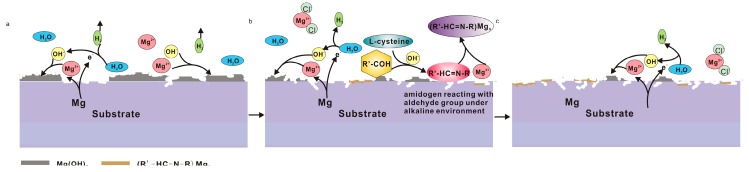
Schematic illustration of the corrosion process of pure Mg during immersion in solution #4: (**a**) I stage; (**b**) II stage; and (**c**) III stage.

Figure 9 illustrates the corrosion mechanism of pure Mg in the saline solution with the presence of amino acids and glucose. In the stage I (Figure 1a and Figure 9a) before an immersion of 8 h, the solution pH value (Figure 1b) was the lowest among the solutions. In the acidic solution (less than 6.0), dissolution of pure Mg was observed and the corrosion rate was enhanced. Consequently, the rapid increase in pH of the solution resulted in alkalinization and reduction in HER (Figure 1a) on the basis of Reactions (1) and (2).

In the stage II (8–32 h of immersion; Figure 1a and Figure 9b), the alkaline solution provided an essential condition for the formation of Schiff base (-C=N-) as shown in Reaction (5). L-cysteine reacting with glucose caused the formation of the -C=N- group. The amount of -C=N- increased with time and the negatively charged molecules chelated with Mg^2+^ ions. Hence, a protective film of Mg(OH)_2_ was very hard to precipitate on the surface (Figure 3h). Simultaneously, Cl^−^ ions (as shown in Figure 6h) preferentially combined with Mg^2+^ ions to form soluble MgCl_2_. The effects from the Schiff base and chloride ions accelerated the corrosion. 

In the stage III (after 32 h of immersion; Figure 9c), the corrosion rate tended to be stable gradually due to the depletion of amino acids in the later stages.

## 4. Materials and Methods

### 4.1. Materials

Commercially available pure Mg ingots with a purity of 99.97%, manufactured by Guangling Magnesium Industry Science and Technology Co. Ltd. (Beijing, China) were utilized for all the tests. They were cut into samples with the sizes of 20 mm × 20 mm × 2.5 mm for the tests. The samples were ground with Al_2_O_3_ abrasive paper to 2500 grit, washed with distilled water and ethanol, and dried with warm air prior to the corrosion tests. The chemicals used in the tests contain sodium chloride, glucose (C_6_H_12_O_6_) and L-cysteine (HSCH_2_CH(NH_2_)COOH) which is an essential component of protein in the human body [47].

### 4.2. Immersion Testing

The solutions are shown in Table 4. A simple saline solution (0.9 wt. % NaCl) was used in the study to avoid the interruption of species such as hydrogen carbonate, sulfate, hydrogen phosphate in Hank’s solution. The concentration (6.0 mg/L) of L-cysteine was chosen based on human blood plasma with a concentration of 50.67 ± 5.01 μmol/L or 5.5–6.7 mg/L [48]. And E. Balint et al. indicated that the content of glucose of human blood plasma is 4.6~6.1 mmol as a normal; 10 mmol/L after a meal; and 15 mmol/L for hyperglycemia population [49]. The immersion test was carried out at 37 ± 0.5 °C. The Mg specimen was put in separate beakers containing the respective solutions [50]. A funnel was placed over the specimen, which was positioned on a polytetrafluoroethylene (PTFE) plate with through holes to ensure the solution was exchangeable and hydrogen collection could occur. In the hydrogen evolution test, the ratio of the solution volume to the sample surface exposed to the solution was 30 mL/cm^2^. Note that, the focus of this study was the corrosion mechanism of pure Mg in the effect of glucose and/or amino acid. The threshold level of the concentration of glucose and/or amino acid to influence the rate of corrosion of Mg should be further explored. The hydrogen evolution rate (HER, *V*_H_) can be characterized as [51]

*V*_H_ = *v*/*St* (6)

where *v* is the hydrogen evolution volume (mL); *S* and *t* are the sample area exposed to solution (cm^2^) and immersion time (h), respectively. 

The volume of hydrogen gas and the pH values of solutions were recorded every hour. The pH values of the solutions were measured using a pH meter (pH400 type). Four parallel samples were prepared for each immersion test. 

### 4.3. Electrochemical Experiments

An electrochemical workstation (PARSTAT 2273) was applied to investigate the electrochemical corrosion behavior of the samples. A three-electrode cell was used with the sample as the working electrode (with an exposed area of 1.0 cm^2^), a saturated calomel electrode (SCE) as the reference electrode and a platinum plate as the counter electrode. Polarization measurements were carried out in the corrosion cell containing 500 mL of solution. Electrochemical impedance spectroscopy (EIS) measurements and Tafel polarization curves were recorded after an immersion delay of 3600 s. EIS measurements were conducted at the frequency ranged from 100 kHz to 10 mHz at open circuit potential by applying 10 mV sine wave AC voltage. The scanning potentials of Tafel polarization curves ranged between −2.0 V/SCE and −1.0 V/SCE at a scan rate of 1 mV/s. Four parallel tests were conducted for each solution. 

Usually, polarization resistance, *R*_p_, which is inversely proportional to the corrosion rate, can be calculated using the simplified Stern-Geary equation [52]

(7) $$R_{p}=\frac{b_{a}\cdot b_{c}}{2.303i_{\mathrm{corr}}(b_{a}+b_{c})}$$

where *b*_a_ and *b*_c_ represent the Tafel slopes of the anode and cathode, respectively. *i*_corr_ is the corrosion current density.

### 4.4. Surface Analysis

The constituents of corrosion products were examined by means of X-ray diffraction (XRD, Riguta D/max-RC, Japan) with a Cu K_a1_ (λ = 0.15406 nm) source operated at 35 kV and 20 mA, Fourier transformed infrared (FTIR, Nicolet380, Thermo Electron Corporation, MA, USA) and X-ray photoelectron spectroscopy (XPS, ESCALAB 250, Thermo VG Corporation, MA, USA). The surface morphologies of the samples were examined by field emission scanning electron microscope (FE-SEM, NOVA NANOSEM-450, FEI Corporation, Portland, OR, USA).

## 5. Conclusions

In the present study, the corrosion behavior of pure Mg in NaCl solution with amino acids (L-cysteine) and glucose is systematically investigated. The following conclusions are made:

(1) The polarization, EIS and hydrogen evolution tests indicate that glucose or amino acids (L-cysteine) delay the corrosion of pure Mg in saline solutions, whereas L-cysteine coupled with glucose enhances the corrosion rate of the samples.
(2) The XPS results demonstrate that the coupling effect of L-cysteine and glucose gives rise to the formation of Schiff base (R’C=N-CH-R) due to the alkalinization of the initial acidic solutions with both amino acids and glucose during corrosion of pure Mg.
(3) The results of SEM, EDS and FTIR show that the formation of (RNH_2_CHCOO)_2_Mg on the surfaces and the depletion of amino acids prevents the further corrosion of pure Mg; hence, the corrosion rate remains stable.
(4) There might be a critical glucose content regarding its influence on corrosion rate of pure magnesium. Further explorations are needed to understand these effects.

## Figures and Tables

**Table 1:** Electrochemical parameters of polarization curves.

Solution	*E*_corr_ (V/SCE)	*i*_corr_ (A/cm^2^)	*b*_a_ (mV/Decade)	−*b*_c_ (mV/Decade)	*R*_p_ (Ω·cm^2^)
#1	−1.56	6.90 × 10^−6^	81.50	245.01	7. 70 × 10^3^
#2	−1.54	4.67 × 10^−6^	129.14	159.56	6.30 × 10^4^
#3	−1.55	5.81 × 10^−6^	112.76	187.84	2.11 × 10^4^
#4	−1.64	4.89 × 10^−5^	130.11	240.77	2.29 × 10^3^

**Table 2:** Fitting results of the EIS spectra.

Solution	*R*_s_ (Ω·cm^2^)	*Y*_0_ (Ω^−1^·cm^−2^·s^−1^)	*n*	*R*_ct_ (Ω·cm^2^)	*Q*_f_ (F cm^−2^)	*R*_f_ (Ω·cm^2^)	*L* (H cm^−2^)	*R*_L_ (Ω·cm^2^)	Chi Square
#1	66.25	1.84 × 10^−5^	0.909	1127	3.80 × 10^−3^	407.7	6.18 × 10^4^	1969	1.7 × 10^−4^
#2	66.15	1.63 × 10^−5^	0.917	1207	3.28 × 10^−3^	1078	7.04 × 10^4^	2584	1.5 × 10^−4^
#3	65.81	1.62 × 10^−5^	0.921	922.70	4.28 × 10^−3^	5470	3.80 × 10^4^	1430	2.6 × 10^−4^
#4	66.62	1.95 × 10^−5^	0.907	644.80	3.94 × 10^−3^	292	2.59 × 10^4^	700.9	5.2 × 10^−4^

**Table 3:** Comparison of the effects of albumin and amino acids (L-cysteine).

Materials	Solutions	*i*_corr_ (A/cm^2^)	*R*_ct_ (Ω cm^2^)	Refs.
Pure Mg	0.8 wt. % NaCl	6.20 × 10^−4^	103	Liu [40]
	0.8 wt. % NaCl + 1 g/L albumin	5.80 × 10^−4^	128	
	0.8 wt. % NaCl + 10 g/L albumin	4.50 × 10^−4^	149	
M1A	SBF	3.62 × 10^−4^	-	Wang [28]
	SBF + 40 g/L albumin	2.81 × 10^−4^	-	
Pure Mg	PBS + 0.1 albumin	(3.53 ± 3.39) × 10^−4^	202 ± 241	Mueller [41]
	PBS + 1 albumin	(0.13 ± 7.56) × 10^−5^	405 ± 469	
	PBS + 10 albumin	(3.73 ± 5.41) × 10^−5^	2117 ± 1509	
	PBS	(7.76 ± 8.81) × 10^−6^	7133 ± 5167	
Pure Mg	0.9 wt. % + 0.006 g/L L-cysteine	4.67 × 10^−6^	1207	present work

**Table 4:** Concentration of the chemicals of the solutions for immersion tests, g/L.

Solution	NaCl	L-Cysteine (HSCH_2_CH(NH_2_)COOH)	Glucose (C_6_H_12_O_6_)
#1	9.0	-	-
#2	9.0	0.006	-
#3	9.0	-	2.0
#4	9.0	0.006	2.0
